# Spatial and seasonal trait selection in dung beetle assemblages along an aridity gradient in the Sahara

**DOI:** 10.1002/ecy.70106

**Published:** 2025-05-14

**Authors:** Indradatta de Castro‐Arrazola, Francisco Sánchez‐Piñero, Marco Moretti, Joaquín Hortal

**Affiliations:** ^1^ Departamento de Ecología, Facultad de Ciencias Universidad de Granada, Campus Fuentenueva Granada Spain; ^2^ Department of Biogeography and Global Change Museo Nacional de Ciencias Naturales (MNCN‐CSIC) Madrid Spain; ^3^ Departamento de Zoología, Facultad de Ciencias Universidad de Granada, Campus Fuentenueva Granada Spain; ^4^ Biodiversity and Conservation Biology Swiss Federal Research Institute WSL Birmensdorf Switzerland; ^5^ Departamento de Ecologia, Instituto de Ciências Biológicas Universidade Federal de Goiás Goiânia Goiás Brazil; ^6^ cE3c – Centre for Ecology, Evolution and Environmental Changes Faculdade de Ciências da Universidade de Lisboa, Campo Grande Lisbon Portugal

**Keywords:** adaptation, aridity, community assembly rules, competition, drylands, environmental filtering, functional responses, resource acquisition strategies

## Abstract

Ecological communities under extreme environments are shaped by a balance of environmental filtering and coexistence mechanisms that result in a series of assembly rules. Although there is abundant evidence about the importance of these community assembly mechanisms in plants, their effects have been seldom compared for animals. We assess their relative importance for the temporal and spatial responses of dung beetle communities along a strong aridity gradient in the edge of the Sahara. Specifically, we study how phylogenetic and functional community structure varies with aridity in space and time and combine it with selected traits to assess the relative importance of mechanisms associated with known assembly rules along the gradient, including whether increasing aridity selects for specific trait values. We surveyed a 400‐km gradient toward the Sahara in the dry and wet seasons of two consecutive years, gathering phylogenetic information and quantifying traits related to aridity from direct measurements and the literature. We calculated metrics of functional and phylogenetic diversity, the decoupled variation in functional diversity, functional and phylogenetic beta diversity, their standardized effect sizes based on null models, and community weighted means for all relevant traits. Then, we assessed the relationships between the spatial and temporal variations in these facets of diversity through linear models, independent principal components analyses, and multiple ANOVAs (MANOVAs). Increasing aridity filters dung beetle communities at the phylogenetic and functional levels, selecting particular trait syndromes in both space and time, as communities change similarly toward the Sahara and between the wet and dry seasons. Contrary to expectations, phylogenetic and functional structure shows a continuous replacement with aridity rather than a nested loss of lineages and trait values, which is not accompanied by a reduction in trait volume along the gradient. Only the hyperarid conditions of the dry season show reductions in trait volume. This implies that responses to aridity lead to assemblages with a common adaptive strategy, dominated by saprophagous species with longer wings and endocoprid behavior, associated with the acquisition of ephemeral resources in the poor desert environment. In addition, animal communities can respond to rapid ecosystem breakdowns if their phenological amplitude includes extreme conditions.

## INTRODUCTION

Understanding how evolutionary constraints determine the diversity and structure of species assemblages requires identifying the general rules driving community assembly. However, the complex nature of ecological communities hampers identifying regularities (Lawton, 1999). Shifting the focus of community ecology toward the interaction between regional communities (i.e., regional species pools) and local assembly of species (Ricklefs, 2008, 2015) may offer an opportunity to find these regularities. Species' functional traits (defined based on Violle et al., 2007) (co)evolve along large spatial and temporal extents (see Thompson, 2005) to form a regional pool of species that are then progressively filtered by dispersal limitations and how local abiotic conditions conform to species' niches and mechanisms of species coexistence (Cornell & Harrison, 2014). This allows conceptualizing communities as the by‐products of a series of general processes that shape the assembly of species into communities (Bennett et al., 2016; Guisan & Rahbek, 2011). Community assembly involves macroecological constraints to the number of individuals and species that can coexist in the community according to the available energy and space (Guisan & Rahbek, 2011) and a series of assembly rules that determine the composition of the species assemblage that persists within the community (Diamond, 1975; Weiher & Keddy, 1995). These community assembly rules include stochastic processes associated with dispersal limitations (which determine which species are able to reach the community) and niche‐driven deterministic responses to the abiotic and biotic conditions of the community, which interact in complex ways (Brousseau et al., 2018; Hortal et al., 2012; Soberón, 2010). While environmental filtering would select for similar niches due to stresses imposed by abiotic variables, the biotic components of the community relate through different coexistence mechanisms, which can either intensify or relax intra‐ and interspecific competition in different ways, resulting in limiting similarity and facilitation processes that select for distinct niches and/or phenotypes to avoid competition for available resources or while benefiting from the facilitator (Mason et al., 2013 and references therein). This creates spatio‐temporal dynamics between equalizing and stabilizing processes (i.e., selecting similar or dissimilar individuals, respectively; Chesson, 2000; HilleRisLambers et al., 2012) as the relative importance of these two main processes varies across spatial and temporal scales (Perronne et al., 2017) according to the strength of the selection by different types of constraints at each particular scale (Vellend, 2010).

Strong stress gradients along environments where resources are scarce provide suitable conditions to assess these general principles (Sundqvist et al., 2013). The greater the range of abiotic stress along an environmental gradient, the stronger will be the selection for specific combinations of trait values (Mason et al., 2013), so variations in selected functional traits within and across species can help in understanding the mechanisms determining the diversity and structure of local communities (Götzenberger et al., 2012; Violle et al., 2014). Communities subject to environmental filtering mechanisms will typically show a progressive narrowing of the variability in the trait values selected by these gradients (i.e., β‐traits sensu Ackerly et al., 2006; Silvertown et al., 2006) toward an optimum (i.e., trait clustering or trait syndrome). However, biotic interactions can lead to trait overdispersion, especially those selected under coexistence (i.e., α‐traits sensu Ackerly et al., 2006; Silvertown et al., 2006), when limiting similarity or other stabilizing mechanisms are preponderant (HilleRisLambers et al., 2012; Pausas & Verdú, 2010). Thus, strong abiotic filters would slow down the pace of competition allowing for the coexistence of functionally similar species, while strong biotic interactions would select for species minimizing trait overlap to escape competition (Brousseau et al., 2018). However, both types of effects may create similar patterns, as both of them can select for the same or correlated traits and/or phylogenetically closer species (Pavoine & Bonsall, 2011). Also, facilitation may increase under high physical stress or consumer pressure (Bertness & Callaway, 1994) and some competitors can have large effects on other species, reducing trait and phylogenetic diversity (HilleRisLambers et al., 2012; Mayfield & Levine, 2010). So it can be expected that some traits will show decreasing variability due to environmental filtering or facilitation, while other traits would increase variability due to niche partitioning.

Water availability stands out as one of the major environmental constraints for biodiversity, especially in warm temperate and tropical regions (Hawkins et al., 2003). Besides limiting ecosystem productivity, surviving in dry environments involves developing specific adaptations to cope with reduced water availability (Boothby, 2019; Heatwole, 1996). Arid ecosystems are also subject to extreme environmental conditions that can include high temperatures, wind desiccation, or soil erosion (Callaway & Walker, 1997; Middleton & Sternberg, 2013), which become particularly harsher during the dry season. These conditions result in a hard soil surface and a generalized deficiency of nutrients, which are also patchily distributed in “islands of fertility” under shrubs and specific microhabitats (Schlesinger et al., 1996). Increasing aridity also transforms community structure and dynamics, changing biogeochemical cycles and ecosystem functioning (Berdugo et al., 2020; Maestre et al., 2016), making deserts ideal systems to study the interplay between environmental filtering and limiting similarity (Lavorel & Garnier, 2002). Stresses along aridity gradients impose multiple severe environmental filters causing a strong selection for species with traits allowing them to cope with harsh conditions and to acquire and process the limited resources available, provoking steep responses of community structure along these gradients (Berdugo et al., 2020). However, such responses are not limited to environmental filtering, as interactions play a major role in arid communities, through both limiting similarity trade‐offs and facilitation processes (Kéfi et al., 2008).

The different strategies used by plants and animals to obtain resources in resource‐poor drylands are likely to result in differences in the assembly and structure of their communities. While plants mobilize nutrients from the soil, animals are able to move, making seeking for resources a competitive process that may select for traits related to resource detection and mobility, particularly in groups exploiting ephemeral resources (Butterworth et al., 2023; Elton, 1949). Indeed, their phenological responses are also different: animals may shift to use different food resources, enter into dormancy, or adapt their life cycles to skip the harsher conditions of the dry season, while plants rely on developing physiological mechanisms against drying out or producing longer‐lasting seeds that can germinate faster. However, most knowledge on community‐level responses of biodiversity to aridity has been developed studying plants or, most recently, biological soil crusts (e.g., Maestre et al., 2016 and references therein). The responses of animal communities to aridity gradients have been less studied, mostly limited to their relationship with plant diversity (e.g., Frenette‐Dussault et al., 2013), grazing (e.g., Chillo et al., 2017), or to the effects of specific assembly rules for taxa with particular adaptations to arid environments, such as ants (e.g., Arnan et al., 2018), rodents (e.g., Brown et al., 2002), or lizards (e.g., Melville et al., 2006). Relatively little is known about how functional traits and phylogenetically structured adaptations drive species assemblage in these extreme environments and how these assembly rules along aridity gradients vary in time and space (but see Brown et al., 2002). Accounting for attributes associated with mobility and resource use can enable understanding the mechanisms behind the potential links between functional traits and both environmental conditions and interspecific interactions (Brousseau et al., 2018). Few studies have investigated how aridity gradients structure arthropod communities and trait‐based approaches are still unevenly used for most arthropod taxa due to a lack of adequate trait data and hypothesis‐based approaches (but see de Castro‐Arrazola, Andrew, et al., 2023). However, standardized protocols are now available for measuring their traits (Moretti et al., 2017) for both studying their community‐level responses and comparisons across taxa (Wong et al., 2019).

In this work, we assess the relative importance of environmental filtering and limiting similarity along an aridity gradient in the edge of the Sahara, the largest hot desert on Earth. To do this, we combine trait‐ and phylogenetic‐based approaches to study the temporal and spatial variations in the assembly of dung beetle communities along a strong aridity gradient in Eastern Morocco, spanning ca. 400 km from Mediterranean semiarid to desert Saharan conditions. Besides water availability, this gradient presents steep variations in other factors associated with aridity, such as trophic resource availability (i.e., dung and other decomposing organic matter), vegetation cover, and soil structure (see de Castro‐Arrazola et al., 2018), making it particularly adequate to assess how dung beetle evolutionary constraints and limited resources determine the assembly rules operating under extreme environments. Scarabaeidae dung beetles exploit ephemeral resource patches (sensu Butterworth et al., 2023) and are good indicators of habitat and environmental changes (Gardner et al., 2008; Spector, 2006). Also, their responses to climate are evolutionarily conserved (Hawkins et al., 2007; Hortal et al., 2011). In general, phylogenetically related dung beetle species share common physiological, morphological, and behavioral adaptations that determine their metabolism and respiration, as well as their thermoregulatory and water conservation strategies (Scholtz et al., 2009). Albeit some close relatives may show different behaviors that allow thermoregulation in extreme conditions (Verdú & Lobo, 2008), entire lineages may show the same climatic limits (Hortal et al., 2011). This allows using phylogenetic data as a proxy for unmeasured traits accounting for dung beetle ecophysiological responses (Calatayud et al., 2021; see Díaz et al., 2013). Further, there is a basic understanding of their trait‐based responses to the environment (de Castro‐Arrazola, Andrew, et al., 2023), in particular to aridity (Castro Sánchez‐Bermejo et al., 2022). And their effects on ecosystem functioning are well known, being involved in key functions in arid environments such as nutrient cycling, secondary seed dispersal, and many others (de Castro‐Arrazola, Andrew, et al., 2023; Nichols et al., 2008).

We use information on phylogenetic relationships and traits related to aridity to answer three specific questions: (Q1) How does dung beetle phylogenetic and functional community structure vary along a severe aridity gradient through space and time? (Q2) What is the relative importance of environmental filtering and limiting similarity in driving community assembly along the gradient? (Q3) Is increasing aridity selecting specific trait values that may result in trait syndromes adapted to extreme aridity? Question Q1 was investigated by analyzing pairwise functional and phylogenetic dissimilarities of dung beetle communities and their partition in turnover and nestedness components along the aridity gradient and between the dry and wet seasons. As Palearctic dung beetles show climatic niche conservatism (Hortal et al., 2011), we expect that equalizing filtering processes will produce phylogenetically and functionally nested assemblages as aridity increases in both space and time (i.e., between seasons). To answer question Q2, we analyzed patterns of functional and phylogenetic standard effect sizes (SESs) of mean pairwise distance (MPD) and mean nearest taxon distance (MNTD) of dung beetle communities along the aridity gradient. Increasingly harsher environments are expected to filter out most potential colonizers, resulting in communities adapted to live in such harsh conditions made up of species with similar traits related to responses to aridity and reduced trait volume (e.g., Mudrák et al., 2016). Therefore, we expect that progressively drier conditions result in an increase in trait and phylogenetic clustering as community assembly is primarily driven by environmental filtering, particularly for MPD. However, limiting similarity is expected to play a significant role in desert areas, where stronger competition for resources will select for ecologically dissimilar species, increasing trait overdispersion for MNTD. The multi‐trait approach of Q1 and Q2 may not enable us to identify trait syndromes adapted to aridity, so in Q3, we assess trait convergence and overdispersion along the gradient through their community weighted means (CWMs), identifying traits that allow species to thrive in the harsh desert conditions and assessing their contribution to the patterns found in Q1 and Q2. This work assesses for the first time the spatial and temporal phylogenetic and functional response of animal communities along a strong aridity gradient, providing novel insights on assembly rules in desert environments.

## MATERIALS AND METHODS

### Study area

We surveyed a ca. 400‐km linear transect following a strong aridity gradient parallel to the Morocco‐Algerian border from the semiarid Mediterranean coast in the north toward the hyperarid (i.e., extremely dry) conditions of the Sahara toward the south (see de Castro‐Arrazola et al., 2018). This gradient is characterized by a threefold difference in annual rainfall (from ca. 350 mm at the semiarid area to 100 mm on the verge of the Sahara). The whole area is under a Mediterranean precipitation regime with a dry season subject to severe summer drought and a wet season in November–March (Belda et al., 2014). For details on the availability of livestock dung, grazing intensity, soil type and vegetation type, height and cover along the gradient, see de Castro‐Arrazola et al. (2018).

### Sampling design

Dung beetles were collected during four survey campaigns: two right after the wet season (April 2013 and 2014) and two in the dry season (September 2013 and 2014). These two seasons represent the two peaks of dung beetle richness and abundance in the Mediterranean region (Hortal & Lobo, 2005). Precipitation was about 20% higher in the wet season of 2012–2013 than in 2013–2014. In each campaign, we surveyed 10 sampling sites, located every ca. 40 km along the transect. Sampling sites were replicated twice, 1 km from each other. Each replicated site consisted of five pitfall traps (11.5 cm diameter) baited with fresh cow dung (thus, 100 traps per sampling campaign) separated 20 m one from another (see survey details and site location in de Castro‐Arrazola et al., 2018). Traps were active for a standard period of 72 h (Amraoui et al., 2016; Labidi et al., 2012), and collected beetles were immediately transferred to 96% ethanol in the field and transported to the lab for species identification, DNA extraction if previously not available, and trait measurement (see details below). In total, we captured 70,326 individuals of 65 dung beetle species in the four sampling campaigns (9627 individuals of 29 Scarabaeinae species and 60,699 individuals of 32 Aphodiinae species), with a slightly higher abundance and richness in 2014 than in 2013 for both seasons (see full dataset at de Castro‐Arrazola et al., 2018, and a summary of species abundances at Appendix S1: Figure S1). Four of these species (*Phalacronotus quadriguttatus*, *Rhyssemus bedeli*, *Rhyssemus vaulogeri*, and *Rhyssemus* new sp.) were represented by just one or two specimens, so most quantitative traits could not be measured (see below) and were discarded from the analyses.

### Phylogenetic data

Phylogenetic information comes from a phylogenetic inference of 202 Scarabaeidae species from the Palearctic (J. Calatayud, J.E. Uribe, N. Guil, I. de Castro‐Arrazola, R. Zardoya, J. Hortal et al., unpublished). Phylogenetic reconstruction was based on one nuclear (28S) and two mitochondrial (COI and COII) markers that were used to conduct a Bayesian inference. Molecular dating was based on five fossil calibration points according to Ahrens et al. (2014). To account for phylogenetic uncertainties, we randomly selected 1000 phylogenetic trees from the posterior distribution after the burn‐in period. These trees (i.e., phylogenetic hypotheses) contained 42 species out of a total of 61 included in the analyses (69%). The 19 missing species (31%) were included in the phylogenetic trees using the SUNPLIN software (Martins et al., 2013). Here, the species are randomly inserted in each tree below the most derived clade where they can be unequivocally assigned based on taxonomic information (Rangel et al., 2015). These random insertions were done 1000 times for each molecular tree. Thus, we obtained 10^6^ trees from which we randomly selected 1000 trees to use in subsequent analyses. The consensus trees for all four phylogenies are available in Appendix S1: Figure S1.

### Trait data

We selected a series of dung beetle traits that are known to respond to aridity (Castro Sánchez‐Bermejo et al., 2022; de Castro‐Arrazola, Andrew, et al., 2023; Verdú & Galante, 2004). These traits relate to individual growth, survival, and reproduction under progressively harsher conditions— drier atmospheric conditions, more scattered and drier dung (their primary food resource), and harder soils to dig into. We expect that each one of these consequences of aridity affects different aspects of dung beetle biology, so the traits involved in their responses to aridity may involve different features of their life cycle: (1) phenology (daily, seasonally, yearly) and physiology responding to atmospheric dryness, (2) trophic preferences and foraging movement responding to reduced dung abundance and distribution, and (3) reproduction–burial habits and the ability to respond to soil hardness. Based on that, we selected traits linked to these aspects of dung beetle biology (see Appendix S1: Table S1): in particular, adult trophic preference; feeding relocation strategy; linear and surface measurements of head, pronotum, abdomen, and elytra; and protibia and metatibia (i.e., fore and hind legs), which are known to be directly related to reproductive dung removal and burial (de Castro‐Arrazola, Andrew, et al., 2023); and wing measurements, which are expected to reflect the ability to disperse and access food resources—which is of high relevance due to the scattered distribution of dung in arid habitats.

All morphological traits were measured with a Leica M165C microscope using Leica Application Suite LAS V4.0 with the Z‐builder module to process the images and obtain the measurements. Not all traits could be measured in all 61 species sampled in the study. Specimens of 11 very rare species (≤2 captured individuals) were used for obtaining molecular data or placed in reference collections. And the bad preservation and/or minute size (i.e., below the detection threshold of the measuring tools) prevented the gathering of any morphological trait data for four species and wing trait data for another two (see https://doi.org/10.20350/digitalCSIC/15225). Adult trophic preferences and dung relocation strategy for feeding purposes (both qualitative traits) were obtained from the literature and expert knowledge. For these categorical traits, values could be assigned with confidence to all species. For continuous traits, we aimed at measuring 10 individuals per species (Bolnick et al., 2011), and finally, 80% of species had measures for 5 or more individuals due to the limited numbers of individuals in the samples (as in Bishop et al., 2015, but see Griffiths et al., 2016). The individuals measured for each species were chosen from as many localities as possible, and also selecting as much variation in body size as possible, to maximize intraspecific trait variation while covering their distribution along the gradient. In total, we measured 23 traits on 347 individuals (mean 5.8 and median 5.0 individuals per species, as in Bishop et al., 2015) leading to a total of more than 7000 measurements, complemented with further categorical traits gathered from literature or our own observations (Appendix S1: Table S2, and database at https://doi.org/10.20350/digitalCSIC/15225).

The uneven distribution of missing traits across taxa could lead to biased results (van der Plas et al., 2017). These effects would be minimal on the abundance‐weighted metrics explained below (MPD, MNTD, and CWM), as only rare species (with one or two individuals) had missing traits. However, they could potentially have an effect on incidence‐based metrics like Sørensen beta diversity (Perronne et al., 2017; Violle et al., 2017), so traits with measurements for less than 80% of the species were discarded, leaving a total of 7 qualitative and 21 quantitative traits used for the analyses.

### Data analyses

**FIGURE 1:**
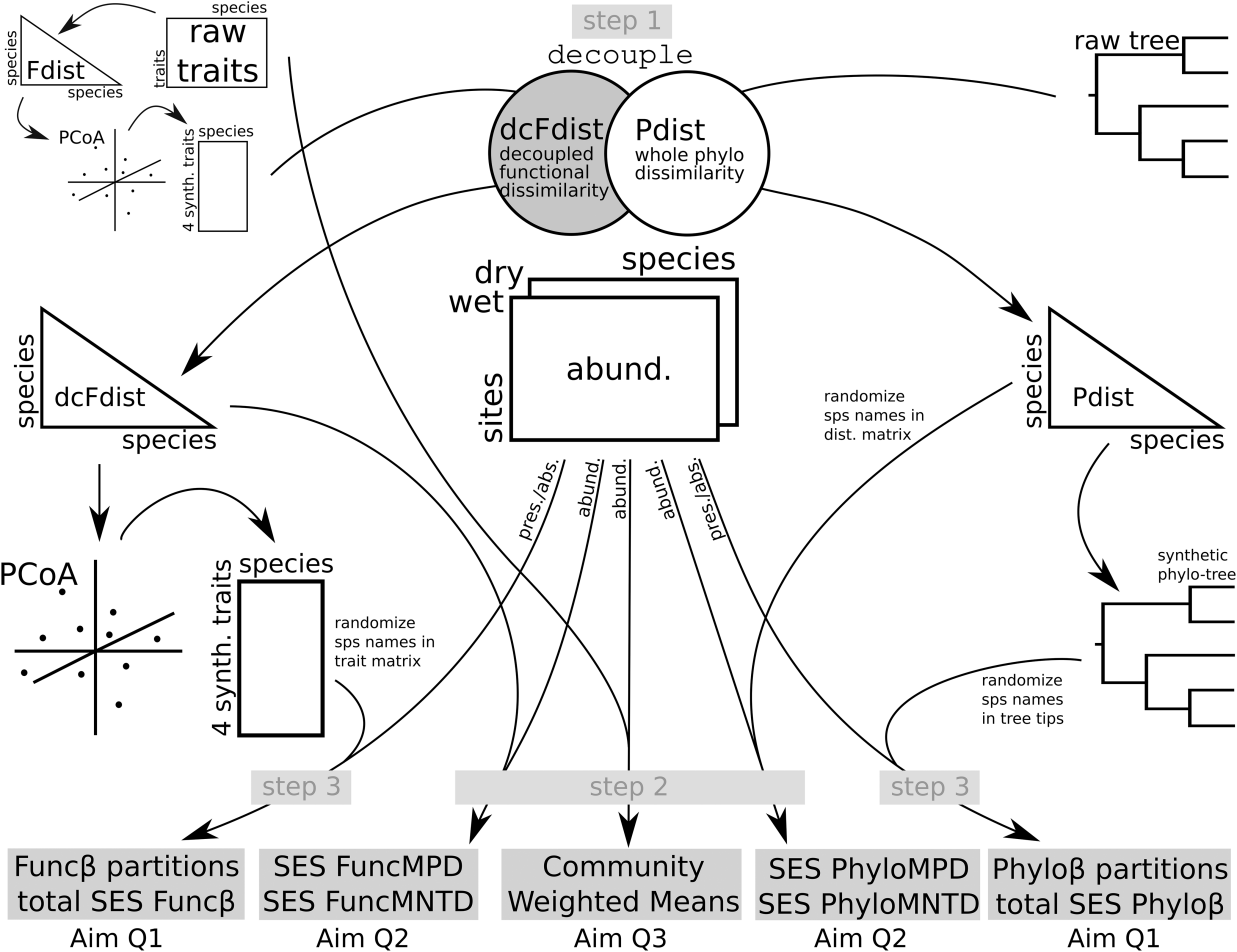
Diagram of the analytical procedure used to answer the questions of the study. For aims Q1 and Q2, the analysis followed the two distinct procedures shown at the two sides of the diagram. The left‐hand side represents the phylogenetic component of dung beetle assemblages; the right‐hand side corresponds to the functional component. In a first step, the phylogenetic signature in the functional data was removed (see de Bello et al., 2017). Then, for each component, similar procedures were followed allowing to calculate the total dissimilarities and their partition into turnover and nestedness components (Aim Q1) and standard effect size (SES) mean pairwise distance (MPD) and mean nearest taxon distance (MNTD) (Aim Q2). The analyses that answer Q3 calculated the community weighted mean (CWM) of specific traits. PCoA, principal coordinates analysis.

To answer our three questions, we conducted separate analyses for the phylogenetic and functional components of dung beetle communities along the aridity gradient in the two distinct seasons (wet and dry). Figure 1 shows the sequence of the analyses, which were similar for the phylogenetic and functional components, although they differed in some steps due to the different nature of the data (i.e., phylogenetic trees and trait matrices) and the algebra used to calculate functional beta diversity. We defined the species pool as all species sampled during a specific season (either dry or wet) along the entire transect. This allows for a finer analysis within each season, as dry and wet seasons have completely different community compositions (de Castro‐Arrazola et al., 2018). Having only one pool would blur any within‐season functional or phylogenetic patterns when comparing observed communities to randomly assembled communities from a species pool that is too widely defined.

#### Step 1: Decoupling phylogenetic and functional variations

Phylogenetic relatedness and trait variation are typically correlated, so phylogenetic differences are often used to account for missing functional trait data in community ecology (but see Díaz et al., 2013), especially in studies disentangling community assembly rules along environmental gradients (see de Bello et al., 2017). However, such correlation implies that analyzing phylogenetic relatedness and trait variation in parallel can lead to partially redundant results. To separate these two components of community variation, we performed a decoupling procedure, which uses matrix residual analyses to separate the functional and phylogenetic components of species dissimilarity (Figure 1). First, we checked that the amount of shared functional and phylogenetic distance (jointFPdist) was small (11%–12% in our communities). Then, we used the R function decouple (de Bello et al., 2017) to divide the dissimilarity between pairs of species within communities into (1) the whole fraction contributed by phylogenetic relationships (Phylogenetic distance, Pdist) and (2) the pure fraction of functional distances solely contributed by traits, independent of phylogenetic relatedness (decoupled Functional distance, dcFdist). Note that this gives priority to phylogenetic over trait differences in the analyses, as only trait variation independent of phylogenetic relatedness is estimated. Although other alternatives separating both independent components of variation have been developed (e.g., Santos et al., 2020), here we opted for providing preeminence to the phylogenetic component because it may summarize the variation in evolutionarily conserved traits for which we lacked data. All distance matrices obtained in the analyses were Euclidean.

#### Step 2: Metrics of phylogenetic and functional diversity and of mean trait values

We used MPD and MNTD to describe both the phylogenetic and functional diversities of each community (Webb et al., 2002) based on the decoupled distance matrices (see Figure 1). With regard to phylogenetic distances, although these two metrics may correlate to some degree, phylogenetic MPD informs about processes selecting species related through deep nodes in the phylogenetic tree, while phylogenetic MNTD does so for the terminal branching of the trees (e.g., congeneric species in the same community) (Kembel et al., 2010), thus providing information about whether assembly rules select species between, respectively, lineages with distinct evolutionary histories or close relatives which are more likely to share common adaptations. A parallel explanation applies to functional MPD and MNTD when considering distances between species in a multi‐trait space; MPD relates to the overall size of the volume occupied by the traits, whereas MNTD describes the degree of filling within such trait volume (see Cadotte & Tucker, 2017; Pavoine & Bonsall, 2011). The four metrics were abundance weighted, as very different proportions of the same species occur in different communities along the gradient, especially during the dry season (de Castro‐Arrazola et al., 2018). Finally, community weighted mean (CWM) was computed for all traits during all four sampling campaigns.

#### Step 3: Assessing changes in community structure along the gradient

To calculate the overlap between pairs of communities, we used functional and phylogenetic beta diversity using the R package betapart (Baselga et al., 2017; see also Branco et al., 2020). To calculate phylogenetic beta diversity, we back‐transformed the Pdist triangular matrix into a synthetic phylogenetic tree that resembles the original tree and combined it with the species incidence matrices. On the functional side, we applied a principal coordinates analysis (PCoA) and used its first eigenvectors as orthogonal synthetic traits. For each campaign (season and year), we selected the appropriate number of synthetic traits limited by the maximum number of dimensions to compute hypervolume overlap. This is defined by both algebraic limitations (max dimensions = 4) and the minimum number of species in any of the compared sites (max dimensions = richness − 1) (Baselga et al., 2017; Villéger et al., 2013). We partitioned total beta diversity (both functional and phylogenetic) into its turnover (i.e., the communities host different functional strategies) and nested components (i.e., one community hosts a small subset of the functional strategies present in the other one) (Villéger et al., 2013), and we calculated the SES (see details below) for total diversity and each of the partitions. In order to present relevant comparisons of the ten sites along the aridity gradient for each campaign, we calculated mean pairwise beta diversity of each site with all others in the gradient (thus, ten mean pairwise beta diversities) and show the selected values of total and partitions of beta diversity between consecutive pairs of sites (thus, nine consecutive beta diversities).

We applied different null models to build virtual communities under stochastic community assemblage rules for both functional and phylogenetic diversities and each species pool (i.e., species sampled in each campaign, see above). To calculate the expected values of phylogenetic metrics, we used a *random tips null model* that accounts for what would phylogenetic diversity be if the species were differently related to each other. To do this, we randomized tip names within the tree of the species pool and then used it in combination with the original and unaltered incidence or abundance species × sites matrix. This method ensures that species richness and abundance distribution remain unchanged between observed and virtual communities. To calculate the expected values of functional metrics, we used a *random traits null model* that responds to what would functional diversity be if the species had different trait values. To do this, we randomized species names among those present in the pool and then used it in combination with the observed incidence or abundance in the species × sites matrix. This method ensures that observed and virtual communities have identical taxonomic richness and abundance distributions, although the resulting null models may potentially assign trait values of dominant species to rare ones (Perronne et al., 2017), which, arguably, corresponds to a scenario with no assembly rules operating at the trait level. All null models were run 100 times, thus obtaining 100 expected values for each functional and phylogenetic metric, from which we calculated avgExp and sdExp, and finally the SES. We calculated SES to evaluate whether the observed functional and phylogenetic diversity metrics depart from what would be expected by a stochastic community assemblage process. The use of SES also removes the effect of richness on functional and phylogenetic diversity indices.

The variations in mean pairwise functional and phylogenetic dissimilarities along the aridity gradient were analyzed separately for each season and year by means of Mantel correlation tests, using pairwise Euclidean distances of aridity between sites, obtained by means of the *vegdist* function of the R package *vegan* (Oksanen et al., 2018). The relationship between dissimilarity matrices and Euclidean distances of aridity was assessed through Mantel tests using Spearman‐rank correlation in the *mantel* function of the R package *vegan*. Significance levels were set by Bonferroni correction. Mantel correlations were also used to analyze the correlations between taxonomic, phylogenetic, and functional beta diversity. Data of taxonomic beta diversity (Bray–Curtis dissimilarity) were obtained from de Castro‐Arrazola et al. (2018). To compare site dissimilarities between seasons of the same year, we used paired *t* tests analyzing pairwise differences between the wet and the dry seasons.

To analyze the relationship between aridity and functional and phylogenetic SES MPD and MNTD variations, we carried out linear models for complete factorial combinations of quadratic effects of aridity (log_10_ transformed), season, and year on mean SES values. We included quadratic effects of aridity on SES MPD and MNTD to account for curvilinear responses to this gradient. Interactions of year with aridity and season did not significantly affect the explanatory power of the models, so year was removed from the analyses, and only aridity, season, and their interactions were included as explanatory variables. Normality and homoscedasticity of model residuals were checked, and heteroscedasticity corrections were used when departures of homoscedasticity of model residuals occurred. As four different models were carried out (functional + phylogenetic × MPD + MNTD), Bonferroni correction was used to set the significance level.

To uncover the variations in trait CWM along the aridity gradient, we used Independent Principal Components nalysis (IPCA; Yao et al., 2012). IPCA was used because the data did not meet the assumption of multivariate normality required by principal components analysis (PCA) to ensure that components are uncorrelated and independent. Like PCA, IPCA seeks to obtain the orthogonal and independent components that better reflect the underlying structure among variables, achieving optimal dimension reduction without loss of essential information. Thus, IPCA is a robust statistical method that combines the advantages of PCA with maximizing the independence of components when data deviate from multivariate normality. IPCA is also adequate to analyze data with a small number of samples and a large number of variables to extract significant biological information (Yao et al., 2012). Variables were scaled prior to the analysis, and the number of components was chosen based on the kurtosis values of components (identifying when a sudden drop occurred) and their proportion of explained variance. Interpretation of the results was based on the loadings of variables in each component. To help in the interpretation of the results, the sparse IPCA (SIPCA) procedure was used to detect the relevant variables in each loading vector. The number of variables to retain in each component was set to five because the proportion of explained variance was similar between the IPCA model with all the variables and the SIPCA model with the reduced number of traits with the same number of components. Since there were important differences between the wet and dry seasons but not between years, analyses were carried out considering the mean CWM values of each trait per site in both years for each season. To analyze the relationship of SIPCA components (response variables) with aridity (explanatory variable), we used multiple ANOVA (MANOVA) for each season. To carry out IPCA and SIPCA, we used functions *ipca* and *sipca* of the *mixOmics* R package (Rohart et al., 2017). Significance levels were Bonferroni corrected.

## RESULTS

### (Q1) How does phylogenetic and functional community structure vary with aridity through space and time?

**FIGURE 2:**
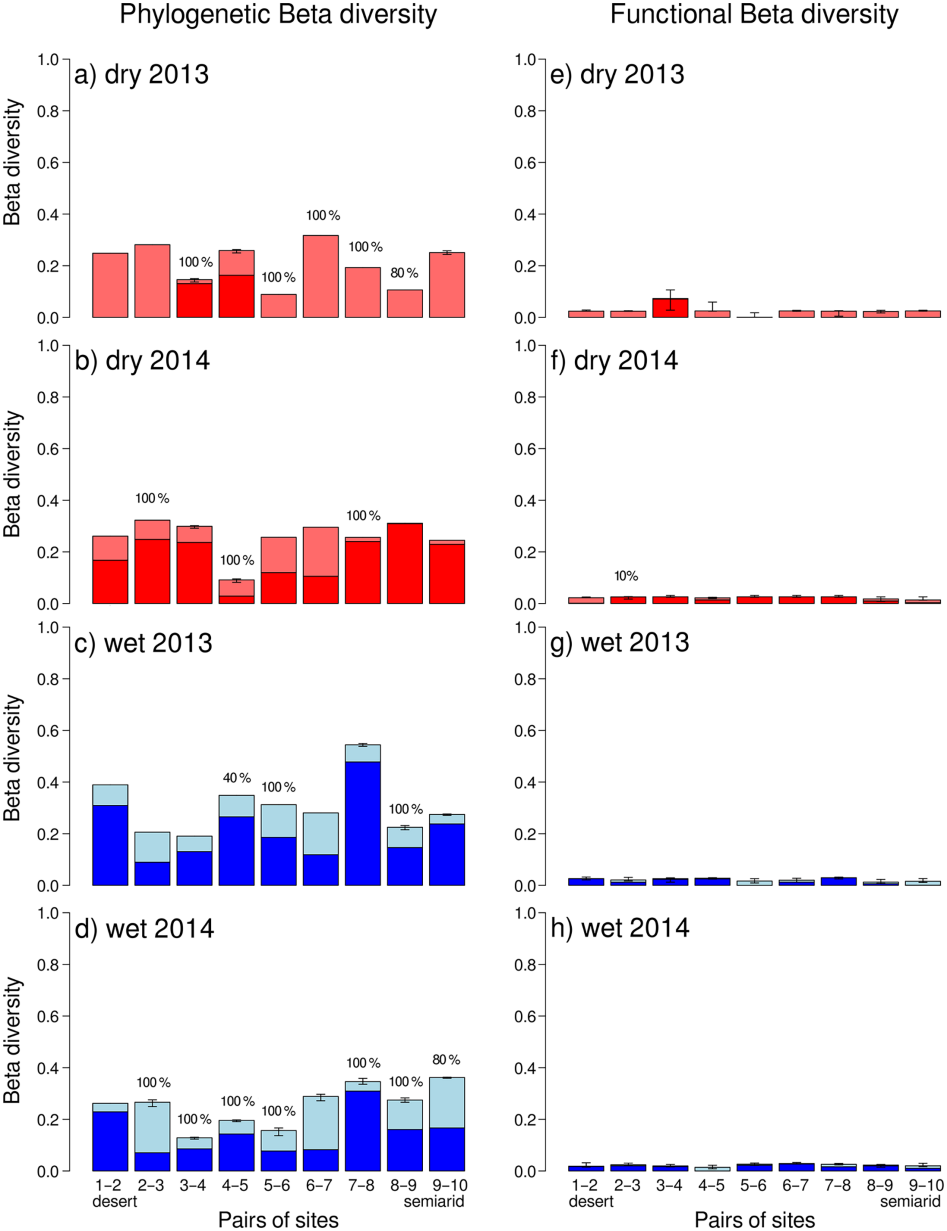
Phylogenetic and functional dissimilarity of dung beetle assemblages between consecutive sites along an aridity gradient from the Mediterranean coast to the Sahara Desert in the wet and dry seasons during two consecutive years (2013 and 2014). Turnover (dark colors) and nestedness (light colors) partitions of beta diversity are shown. Error bars represent the SEs of results due to phylogenetic uncertainty (more than one phylogenetic tree used) which also affected functional results via the decoupling process.

Phylogenetic diversity increased with increasing aridity in both the wet and dry seasons, although more strongly in 2014 than in 2013 (when the dry season showed only marginally significant differences) (Appendix S1: Table S3). Phylogenetic beta diversity was higher in the wet season than in the dry season in both years (wet vs. dry 2013: *t* = −6.6538, df = 44, *p* < 0.0001; wet vs. dry 2014: *t* = −3.3663, df = 44, *p* = 0.0016; paired *t* test) (Figure 2a–d), indicating an overall clustering of lineages during the harsher dry season. In the dry season of 2013, most phylogenetic beta diversity between consecutive sites was due to nestedness (Figure 2a,e). This points to the presence of a few selected lineages along the whole gradient in this season perhaps due to the comparatively lower abundance and richness in this season and year. This contrasts with the much larger proportion of turnover (ca. 75%) observed during all the other sampling campaigns pointing to a higher diversity of lineages. Taxonomic and phylogenetic beta diversities were highly correlated in all sampling campaigns (Appendix S1: Table S4). The decoupled functional dissimilarity matrices obtained were very unstable, making functional beta diversity results strongly unreliable, as most of the structured variation between communities was already captured by phylogenetic beta diversity. Nonetheless, results suggest that functional beta diversity was much smaller than phylogenetic beta diversity in both seasons, contributing only 23%–29% to the total dissimilarity (Appendix S1: Figure S2).

### (Q2) What is the relative importance of environmental filtering and limiting similarity in driving community assembly along the aridity gradient?

**FIGURE 3:**
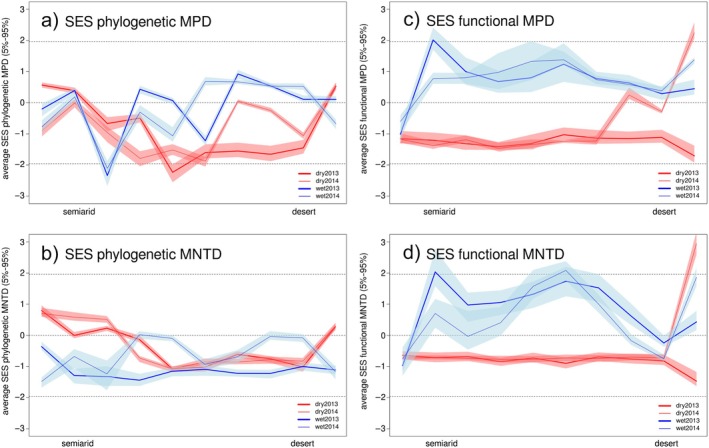
Phylogenetic and functional mean pairwise distance (MPD) and mean nearest taxon distance (MNTD) of dung beetle assemblages along an aridity gradient from the Mediterranean coast to the Sahara Desert in the wet and dry seasons during two consecutive years (2013 and 2014). Shaded areas correspond to 95% CIs due to phylogenetic uncertainty (more than one phylogenetic tree used) affecting functional results via the decoupling process. SES, standard effect size.

The four sampling campaigns showed mostly negative values for phylogenetic SES MPD, indicating a general trend for phylogenetic clustering (Figure 3a). There were neither significant effects of aridity nor season on phylogenetic clustering along the gradient (Table 1). Phylogenetic MNTD shows patterns of both clustering and overdispersion of the lineages present in each assemblage (Figure 3b). Interestingly, the dry season shows nearly random selection (SES values near 0), with a trend to positive SES values toward both the semiarid and hyperarid ends of the gradient (as shown by the significant positive curvilinear effect of the aridity^2^ × dry season term; Table 1), indicating that during the harsher period these assemblages present only distantly related species. This contrasts with the generalized clustering observed in the wet season, when more congeneric species coexist locally, reaching significant SES values for some of the phylogenetic trees (see percentages in Figure 3b).

**TABLE 1:** Results of lineal models relating phylogenetic and functional standard effect size (SES) mean pairwise distance (MPD) and mean nearest taxon distance (MNTD) with quadratic aridity effects (aridity, aridity^2^), season (wet vs. dry), and their interaction (aridity × season, aridity^2^ × season).

Term	df	Phylogenetic			Functional		
		Estimate ± SE	*t*	*p*	Estimate ± SE	*t*	*p*
MPD							
Intercept	1	−0.179 ± 0.183	−0.981	0.3334	0.735 + 0.166	4.432	<0.0001***
Aridity	1	1.968 ± 1.156	1.701	0.0980	0.602 + 1.417	0.425	0.6737
Aridity^2^	1	−0.274 ± 1.156	−0.237	0.8142	−2.051 + 1.385	−1.482	0.1477
Season (dry)	1	−0.620 ± 0.259	−2.398	0.0221	−1.705 + 0.281	−6.060	<0.0001***
Aridity × season (dry)	1	−2.036 ± 1.635	−1.245	0.2218	1.990 + 2.648	0.752	0.4575
Aridity^2^ × season (dry)	1	4.061 ± 1.635	2.483	0.0181	3.765 + 2.489	1.512	0.1397
MNTD							
Intercept	1	−0.884 + 0.130	−6.810	<0.0001***	0.725 + 0.233	3.116	0.0037
Aridity	1	0.337 + 0.899	0.375	0.7103	0.302 + 1.945	0.155	0.8777
Aridity^2^	1	−0.270 + 0.906	−0.298	0.7675	−2.955 + 1.732	−1.706	0.0971
Season (dry)	1	0.553 + 0.156	3.541	0.0012**	−1.322 + 0.346	−3.819	0.0005**
Aridity × season (dry)	1	−2.162 + 1.072	−2.018	0.0516	1.102 + 3.207	0.344	0.7332
Aridity^2^ × season (dry)	1	3.297 + 1.049	3.143	0.0035*	4.531 + 2.953	1.534	0.1342

*Note*: Significance levels after Bonferroni correction: *p < 0.0125; ** *p* < 0.0025; ***p < 0.0001.

Functional MPD differed between the dry and wet seasons, consistently in both sampling years (Table 1, Figure 3c). Dry season communities present significant negative values along most of the gradient, indicating a strong environmental filtering selecting similar trait values in each locality, especially in the intermediate and semiarid sites. By contrast, the wet season shows high positive SES values in several semiarid sites (sites 4, 5, and 9). Functional MNTD shows similar patterns (Table 1, Figure 3d), although SES values show even larger fluctuations in the wet season than in the dry season. During this period, trait volume is relatively constant along the gradient (i.e., similar MPD), but in some sites, species with intermediate trait values are missing (i.e., lower MNTD), leaving gaps within such volume and lowering the density of occupancy of the trait space.

### (Q3) Is increasing aridity selecting for specific trait values?

Kurtosis indicated that only two IPCA components were necessary to describe the CWM structure of assemblages in both seasons. The two components explained 89% of variability in the wet season and 92% in the dry season. The SIPCA including the five most relevant traits of each component explained 87% and 89% of the variation in the wet and dry seasons, respectively, indicating that a few traits were driving assemblage variations (Table 2). The most important factors structuring dung beetle assembly along the gradient were aridity and traits related to the endocoprid and paracoprid feeding strategies (Appendix S1: Figure S3). The first SIPCA component was significantly related to aridity in both the wet and dry seasons (Appendix S1: Table S5), showing that this factor consistently determines the functional structure of the assemblages throughout the whole year. In the wet season, wing length, endocoprid behavior, elytra length, and saprophagy (all Aphodiinae‐related traits) were positively related to aridity, while metatibia area (related to paracoprid behavior, to assist burrowing) was negatively related to it (Table 2). In the dry season, the significant relation of component 1 with aridity indicates that necrophagy, elytra length, endocoprid behavior, and saprophagy were negatively related to aridity and protibia area was positively related to it (Table 2). Interestingly, these results show a contrasting relationship between some of the traits (elytra length, endocoprid behavior, saprophagy) and aridity in the wet and dry seasons (Appendix S1: Figure S3).

**TABLE 2:** Loadings of the different traits in each component of sparse independent principal components analysis for the wet and dry seasons.

Trait	Wet season		Dry season	
	Component 1	Component 2	Component 1	Component 2
Elytra length	0.0149	−0.0034	−0.0476	0.0000
Elytra width	0.0000	0.0000	0.0000	0.0042
Endocoprid	0.0313	0.0000	−0.0468	0.0000
Metatibia area	−0.0023	0.0000	0.0000	0.0000
Necrophagy	0.0000	0.0000	−0.0794	0.0000
Paracoprid	0.0000	0.0045	0.0000	0.0000
Protibia area	0.0000	0.0000	0.0012	0.0000
First protibia tooth length	0.0000	−0.0123	0.0000	0.0000
Saprophagy	0.0104	−0.0037	−0.0052	−0.0068
Wing area	0.0000	−0.0114	0.0000	−0.0026
Wing length	0.0513	0.0000	0.0000	0.0090
Wing load	0.0000	0.0000	0.0000	0.0036

## DISCUSSION

Our results show that aridity shifts the phylogenetic and functional structure of communities via assembly rules acting at the species and trait levels. This is true in both space and time, as communities change similarly toward the hyperarid conditions of the Sahara and in the dry season. Contrary to our expectations (Q1), there is a continuous replacement of the phylogenetic and functional structure of the dung beetle communities with aridity rather than a nested loss of lineages and trait values. That is, the position of the volume of phylogenetic and functional space occupied by dung beetle communities shifts along the gradient, evidencing that varying assembly rules select for different lineages and traits as aridity increases. However, such change in position is not accompanied by a progressive contraction of the overall trait volume of the communities with aridity, thus refuting our expectations that the strong filtering process toward increasingly harsher environments would result in more packed communities composed of species with similar traits and adaptations (i.e., higher trait and phylogenetic clustering, Q2), which is only partially true for the phenological variations, as the pervasive arid conditions along the entire transect during the dry season increased trait clustering. Strikingly, aridity‐driven spatial and seasonal changes in community structure are determined by a combination of assembly rules that select for saprophagous species with longer wings and endocoprid behavior (Q3). This trait syndrome may encapsulate the most important adaptations permitting certain dung beetle species to endure the extreme conditions and acquire the scarce and ephemeral resources available in the hyperarid regions and dry periods of the year.

Aridity proved to be a strong driver of community assembly in both space and time. Taxonomic, functional, and phylogenetic diversity are expected to decrease with aridity due to the strong environmental filtering (e.g., Andriuzzi et al., 2020; Castro Sánchez‐Bermejo et al., 2022; Matías et al., 2018; but see Polis, 1991). However, trait replacement is also found if the range of gradient conditions is wide enough (e.g., Berdugo et al., 2020; Chozas et al., 2017). This is the case of the North‐African dung beetle communities studied here, which show clear shifts in the phylogenetic composition of communities along the aridity gradient due mostly to replacement processes associated with the effect of community assembly rules (see below). This replacement also occurs at the taxonomic level, indicating a change in the dominance of life history strategies as different subfamilies replace one another along the gradient (de Castro‐Arrazola et al., 2018).

Such taxonomic, phylogenetic, and functional replacement along the gradient determines the lack of relationship of phylogenetic and functional clustering with aridity. Abundance shifts from Scarabaeinae to Aphodiinae‐dominated communities as aridity increases (de Castro‐Arrazola et al., 2018), so shifts in the position of the phylogenetic space they occupy may reflect the replacement of species between lineages of these two subfamilies. The limited variations in the size of such space may be because the low phylogenetic resolution within these two speciose clades (Cabrero‐Sañudo & Zardoya, 2004; Villalba et al., 2002) leads to similarly sized mean phylogenetic space and clustering. Because dung exploitation strategies are largely conserved in both lineages, phylogenetic and functional aspects followed a similar overall pattern. Indeed, other dung beetle desert communities show weak effects of aridity on functional diversity (Castro Sánchez‐Bermejo et al., 2022). Only during the dry season there was some phylogenetic overdispersion in both extremes of the gradient, especially at the semiarid end. Overdispersion has been related to changes in plant communities and land use intensification in ant assemblages (e.g., Arnan et al., 2018; Frenette‐Dussault et al., 2013), so our results can be interpreted as fewer species filling the phylogenetic space due to the effects of aridity (at the desert end of the gradient) and disturbance (at the semiarid extreme of the transect) because of agricultural and urbanization transformation of these areas (de Castro‐Arrazola et al., 2018).

Strikingly, the major shifts in both the position and volume of dung beetle functional and phylogenetic diversity occur seasonally. The contrasting shifts in community structure between the wet and dry seasons highlight that the trophic plasticity and rapid phenological responses that life history adaptations confer to animals may be following the same principles as the spatial and long‐term successional responses to aridity shown by plant communities. Using a space‐for‐time equivalence (as in Sundqvist et al., 2013), in the dry season there is a homogenization of the conditions along the whole aridity gradient, which exhibits a desert‐like community. Bishop et al. (2015) observed a similar pattern in ants at a montane ecosystem. Indeed, the small functional volumes found in the dry season suggest the existence of strong constraints that inhibit the successful development of strategies capable of thriving during the harsh season. This indicates the probable existence of community assembly rules related to both severe climatic conditions and scarce resource availability. According to these rules, the spatially sparse and temporally brief availability of short‐time moist dung and the dry compacted soil may impose a strong filtering process that progressively selects for fewer (larger phylogenetic MNTD, especially in the extremes of the gradient), different species (i.e., taxonomic turnover) from the same lineages (same phylogenetic MPD volume) and with similar trait values (small functional volumes).

The progressive functional and phylogenetic replacement toward desert conditions and functional clustering during the dry season are associated with a strong selection for three key traits (trophic preference, nesting strategy, and mobility). These traits compose a trait syndrome favored by several strong assembly rules operating in desert environments. On the one hand, facultative saprophagous species with longer wings can survive exploiting the ephemeral resource patches available in harsh desert conditions (see Butterworth et al., 2023). Arid environments are poor in palatable dung, so many species in arid regions are either saprophagous, being able to digest dry matter in absence of fresh dung (Holter & Scholtz, 2011), or have a wide trophic plasticity (F. Sánchez‐Piñero, personal observations). Saprophagous species are well represented along the studied gradient, and their abundance increases toward the harsher conditions, reaching half of the individuals in all communities during the dry season. On the other hand, this response to aridity may also be related to the trait syndrome shared by most Aphodiinae that includes both the small size associated with endocopry, which enables them to thrive on small droppings. Altogether, this syndrome allows feeding when palatable dung is absent, but also an efficient flight based on long wings, which allows them to reach sparse droppings by performing high‐endurance foraging flights (see Pessôa et al., 2023; Raine et al., 2018).

The importance of wing length for dung beetle survival under conditions of scarce food resources, such as in deserts or heavily humanized landscapes, may be related to its effects on flight ability (de Castro‐Arrazola, Andrew, et al., 2023). Although little is known about the drivers of wing morphology variation and its effect on flight performance in dung beetles (but see Verdú et al., 2012), this trait is related to dung beetle responses to land use changes (Pessôa et al., 2023; Raine et al., 2018) and responds to environmental conditions and affects performance in other taxa (e.g., Berwaerts et al., 2002; Frazier et al., 2008). Lower wing loadings (i.e., larger wings relative to body mass) have lower flying costs and a longer endurance (Gibb et al., 2006; Gyulavári et al., 2014). If this can be extrapolated to dung beetles in search of dung, finding sparser and more ephemeral palatable dung would favor species with longer wings during the dry season and in the most arid sites of the transect.

Our results emphasize the role of traits related to finding and using resources in community structuring and are consistent with varying assembly rules related to productivity (resource) gradients. Although abiotic filtering has been claimed to dominate over competition in stressful environments (Coyle et al., 2014; Weiher & Keddy, 1995), the traits identified in our study indicate a shift of competitive ability along the studied transect derived from changes in productivity, coupled with variations in the importance of dispersal limitations. Changes in abiotic conditions along aridity gradients (increase in temperature, decrease in water availability, higher unpredictability) determine a decrease in productivity and lower resource predictability as aridity increases, shifting the importance of both deterministic and stochastic assembly rules associated with environmental filtering but also with competition and dispersal. In our study, dung availability and plant cover were 4.6–10 times higher in the semiarid than in the Saharan end of the transect, reflecting large differences in primary productivity and resource availability along the aridity gradient, which have also been related to a decrease in species richness (de Castro‐Arrazola et al., 2018). Further, types of dung also differed between the semiarid and arid extremes: while large cattle dung pats were largely available in the semiarid zone, small and drier sheep and goat droppings constituted most of the dung in the desert areas. Moreover, dung in arid areas dries out faster (González‐Megías & Sánchez‐Piñero, 2003; Lumaret, 1995; Vliet et al., 2009), with the consequent enhancement of its already ephemeral palatability and sparse spatial distribution (Lange, 1969). Thus, during the wet season, traits related to higher trophic generalism, including largely saprophagous species, and vagility (flying ability) are selected to cope with limited, sparser, and more ephemeral resource availability (Körtner et al., 2019; Polis, 1991).

Interestingly, traits and aridity show contrasting relationships in the wet and dry seasons. As desert conditions prevail along the entire gradient during the dry season, the higher abundance of dominant Aphodiinae on its semiarid end determines the negative relationship between trait variation and increasing aridity. The strength of the assembly rule associated with resource availability may also be driving this pattern, as the presence of cows and the higher abundance of plant detritus promote the dominance of *Anomius baeticus* (de Castro‐Arrazola et al., 2018). These results are in agreement with previous studies emphasizing the role of productivity in determining community assembly along environmental gradients in both space and time (Brun et al., 2019; Coyle et al., 2014; Zhang et al., 2019).

To summarize, dryland communities are subject to a strong environmental filter toward the most arid conditions of the Sahara, which selects a smaller number of species in both space and time. But rather than being a subset of the richer communities from the wet season and the semiarid areas, desert communities constitute a distinct pool of species from a few unique lineages that are adapted to the harsh Saharan conditions. This evidences the existence of distinct community assembly rules associated with both environmental filtering and the competition for ephemeral resources in drylands. In the localities and seasons subject to extreme conditions, dung beetle communities are dominated by species showing a trait syndrome that includes facultative saprophagy, endocopry, and longer wings. Such particular selection of traits suggests that resource acquisition is one of the main constraints of animal life in the desert, however acting as an equalizing filter selecting traits adapted to harsh desert conditions rather than promoting niche differentiation (limiting similarity), as there is a limited array of body plans that allow being efficient in the desert. So, although overall trait volume may not vary with aridity, the abovementioned trait syndrome is selected by a strong filter that shifts the position of the functional space occupied by communities, creating an assembly rule related to differences in resource availability and predictability. Interestingly, the studied dung beetles showed this shift also seasonally, a temporal component in the response to aridity that shows that animals can respond rapidly to conditions of ecosystem breakdown—a novel and unexpected result that deserves further exploration. In any case, our results evidence that although some general principles coming from the study of plant or microbial communities may apply to animals, their basic differences in life histories and modes of resource acquisition result in distinct community dynamics under extreme arid conditions, particularly in phenological responses and the relative roles of equalizing and stabilizing mechanisms under varying assembly rules. It follows that further studies are needed to understand the commonalities and differences in the responses of plant and animal communities to aridity, as well as the contributions to ecosystem functioning of these trophic levels through space and time.

## FUNDING INFORMATION

Spanish Agencia Estatal de Investigación projects CGL2011‐29317, PID2022‐140985NB‐C21 funded by MCIN/AEI/10.13039/501100011033/FEDER, EU.

## CONFLICT OF INTEREST STATEMENT

The authors declare no conflicts of interest.

## Supporting information


Appendix S1.

## Data Availability

All distributional and abundance data are already published by de Castro‐Arrazola et al. (2018) and are publicly available at https://doi.org/10.7717/peerj.5210. Trait data (de Castro‐Arrazola, Sánchez‐Piñero, et al., 2023) are available in the repository DIGITAL.CSIC at https://doi.org/10.20350/digitalCSIC/15225.
