# Supplementary material for: Spatial and seasonal trait selection in dung beetle assemblages along an aridity gradient in the Sahara
Source: Ecology. 2025 May 14;106(5):e70106. doi: 10.1002/ecy.70106 (PMC12079014; doi:10.1002/ecy.70106)
Supplement: Supplementary file 1 — Appendix S1. [file ECY-106-e70106-s001.pdf]

Spatial and seasonal trait selection in dung beetle assemblages along an aridity gradient in the Sahara. Indradatta deCastro-Arrazola, Francisco Sánchez-Piñero, Marco Moretti, and Joaquín Hortal. *Ecology*.

APPENDIX S1

This file contains the following supplementary materials:

Supplementary Figures S1 to S3

Supplementary Tables S1 to S5

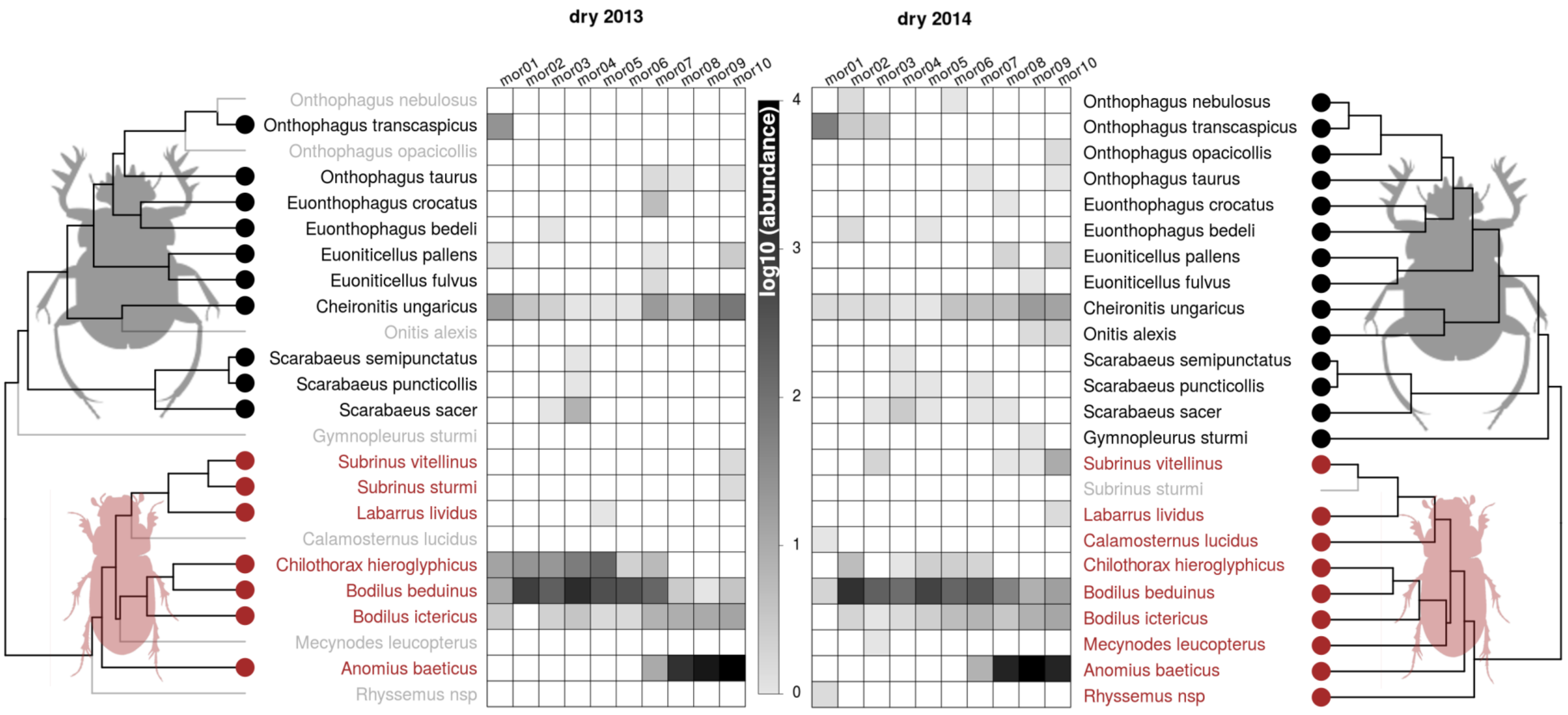

Figure S1. Summary of abundances of species in each of the four sampling campaigns (dry and wet seasons of 2013 and 2014) and their phylogenetic relationships. For each campaign we present phylogenetic relationships using the following codes: Scarabaeinae species - black circles and names; Aphodiinae species - brown circles and names; and c) absent species - grey branches, no circles and grey names. Grey intensity refers to log10(abundance) for each species and site; thus absent species have all white squares in the matrices, while species present at least, one site show some degree of grey shading. Insect silhouettes by Indradadatta deCastro-Arrazola, based on pictures of *Scarabaeus sacer* and *Aphodius foetidus* taken by himself from individuals captured in this work. (Figure continues below)

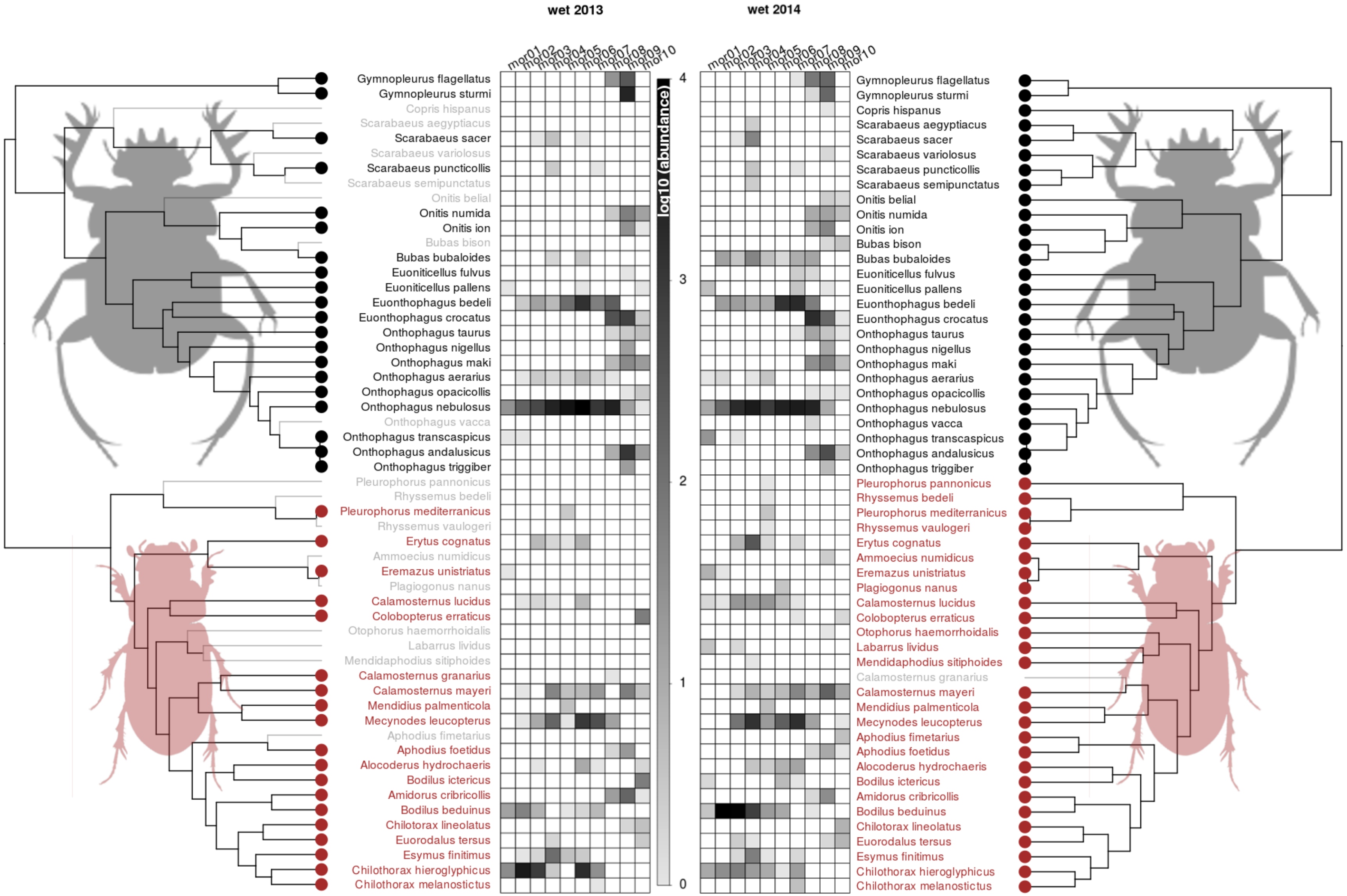

Figure S1. (continued)

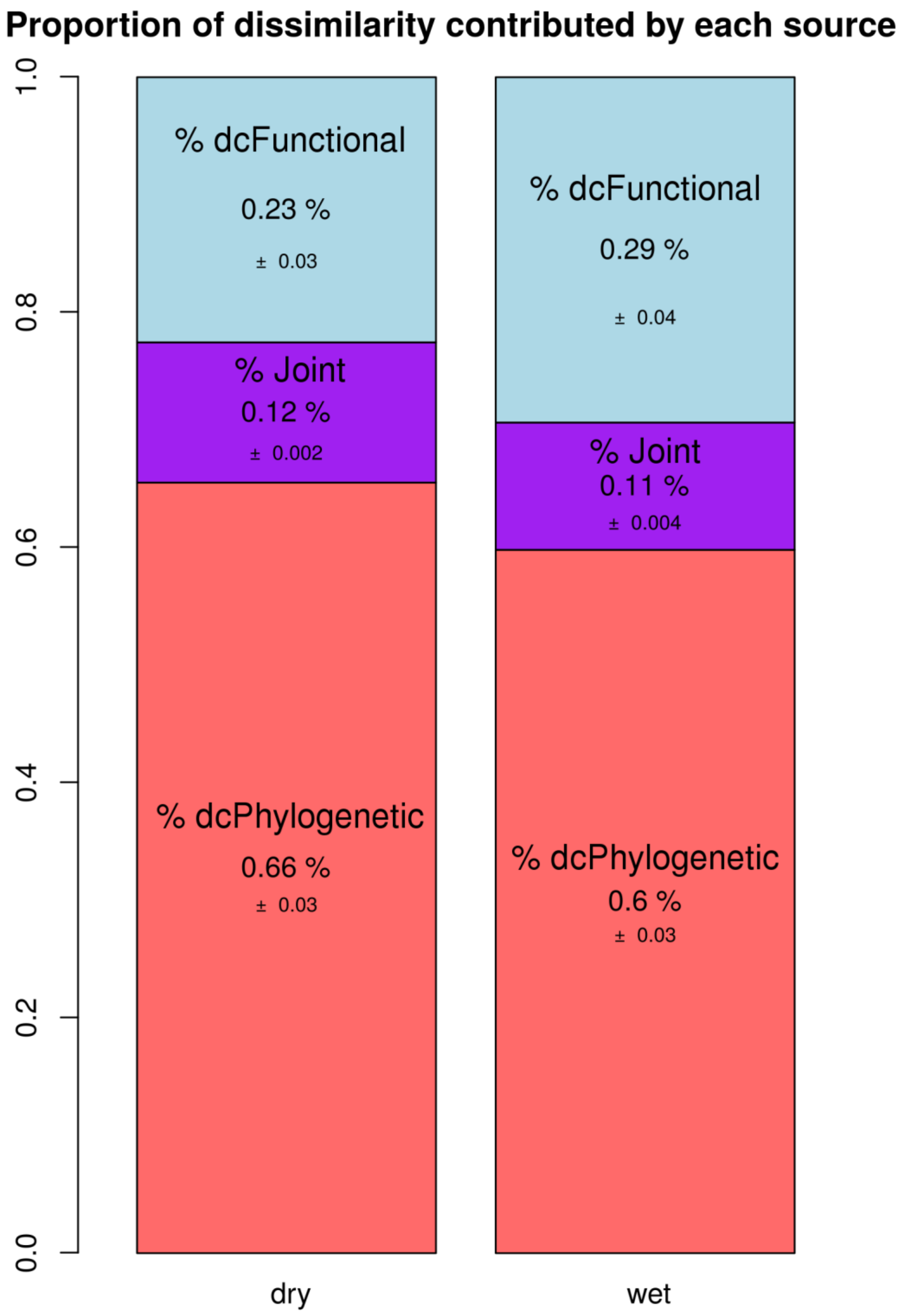

Figure S2. Pure and shared proportions of functional and phylogenetic contribution to the overall dung beetle dissimilarity in two regional pools (wet and dry season) along an aridity gradient of c. 400 km from the Mediterranean Sea to the Sahara Desert. Standard error of results due to phylogenetic uncertainty (100 phylogenetic trees were used) are shown under the percentage of each portion.

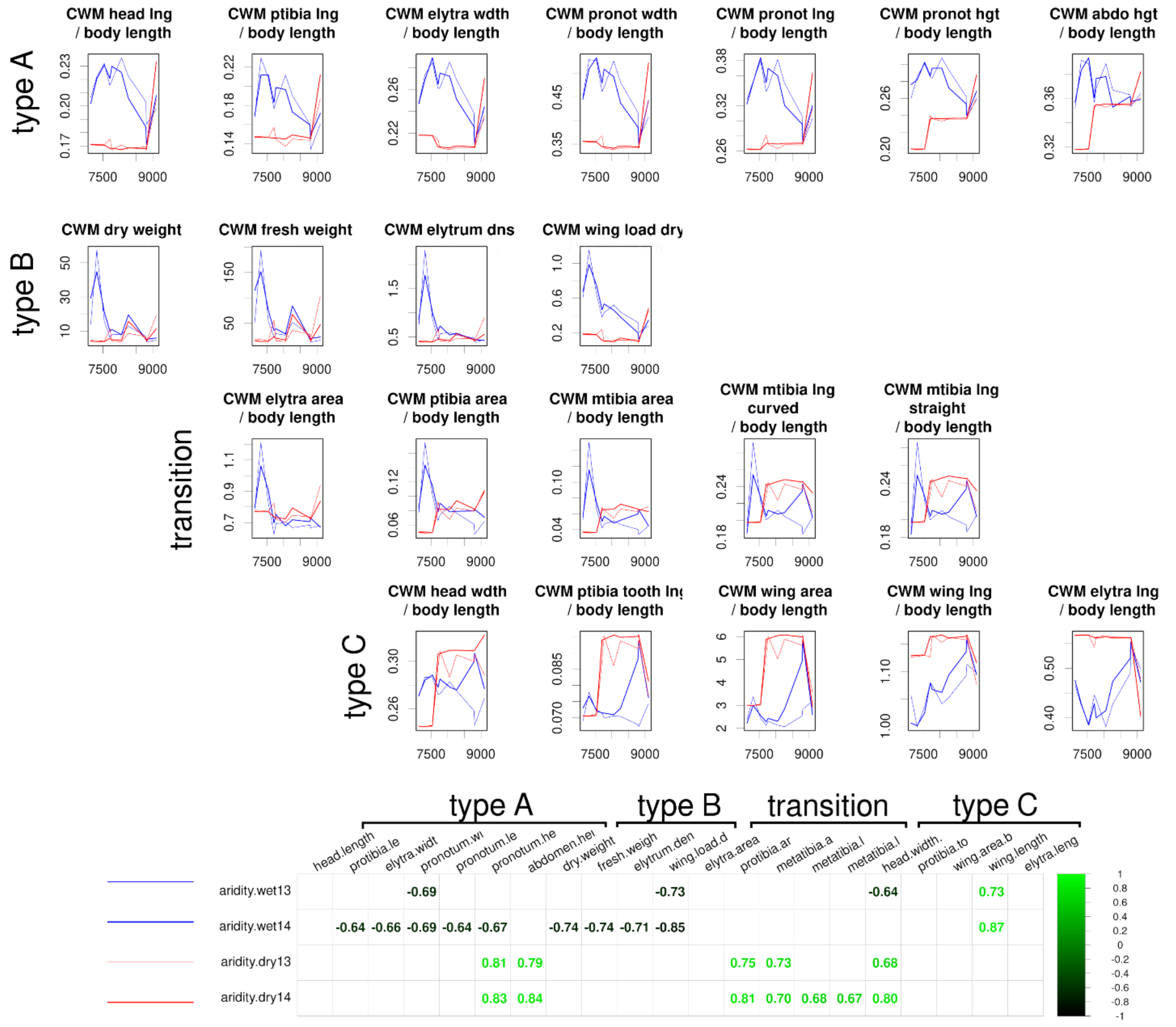

Figure S3. Patterns and correlations of Community Weighted Mean of all quantitative traits measured in dung beetle communities in the wet and dry seasons during two consecutive years along an aridity gradient from the Mediterranean Sea to the Sahara Desert. Three types of traits are proposed according to their pattern along the aridity gradient.

Table S1. Description of trait measurements. Categorization as *reproduction* traits is just a hypothesis based on the literature (see also deCastro-Arrazola et al. 2023). Fuzzy coding of qualitative traits allows reflecting intraspecific variability and/or plasticity by assigning percentages to several categories for each species. All quantitative traits were measured from stereoscope images (see main text for details).

**Trait**	**Category**	**Unit**	**Measurement description or factor levels**
Feeding relocation strategy	Feeding	Qualitative, fuzzy coded	Endocoprid, paracoprid and/or telecoprid
Adult trophic preference	Feeding	Qualitative, fuzzy coded	Saprophagous, coprophagous, micophagous and/or necrophagous
Dry biomass	Morphological	Quantitative	Weight of whole individual after drying in oven at 60ºC for 72h
Fresh weight	Morphological	Quantitative	Weight of whole individual directly from alcohol storage, gently dried on the outside with a paper tissue
Body length	only used for calculations		Sum of Head length + Pronotum length + Elytra length
Head length	Morphological	Quantitative	Distance from clypeus to vertex (no horns included)
Head width	Morphological	Quantitative	Maximum head width, normally at genas
Pronotum height	Morphological	Quantitative	Maximum distance between pronotum discus to point between protibia coxas
Pronotum length	Morphological	Quantitative	Distance along the individuals longitudinal axis
Pronotum width	Morphological	Quantitative	Maximum pronotum width
Abdomen height	Morphological	Quantitative	Maximum abdomen height
Elytrum area	Morphological	Quantitative	Area measured with polyline tool to outline the whole elytrum
Elytra length	Morphological	Quantitative	Distance along the longitudinal axis, from insertion (scutellum included) to apex (pygidium not included)
Elytra width	Morphological	Quantitative	Maximum elytra width, normally at the callus humeralis
Elytrum weight	Morphological	Quantitative	Weight of single elytrum (cut off from joint) after drying in oven at 60ºC for 72h
Protibia area	Reproduction	Quantitative	Area measured with polyline tool to outline the whole protibia
Protibia length	Reproduction	Quantitative	Distance from joint with profemur to insertion of tarsi
Protibia tooth length	Reproduction	Quantitative	Distance from insertion of spine to apex of first tooth of protibia
Metatibia area	Reproduction	Quantitative	Area measured with polyline tool to outline the whole metatibia
Metatibia length straight	Reproduction	Quantitative	Distance from insertion with metafemur to insertion of tarsi in a straight line
Metatibia length curved	Reproduction	Quantitative	Distance from insertion with metafemur to insertion of tarsi following the curvature of metatibia
Wing area	Dispersal	Quantitative	Wing (cut off from joint) area measured with polyline tool to outline the whole wing
Wing length	Dispersal	Quantitative	Maximum distance from joint to wing apex
Wing load	Dispersal	Quantitative	Wing area / dry biomass

Table S2. Summary of trait values for Aphodiinae and Scarabaeinae dung beetle species in the wet and dry seasons during two consecutive years along an aridity gradient from the Mediterranean sea to the Sahara desert. Traits were gathered from literature and field observations (Feeding relocation strategy and Adult trophic habits) and measured values (quantitative traits: all other traits) (see Table S1 for the description of each trait). Column *n* refers to the number of species for which the trait could be measured, *min* to minimum value in any measured species, *mean* is the average value for *n* species (non weighted), *sd* is standard deviation and *max* is maximum value for any measured species.

		**Scarabaeinae**					**Aphodiinae**				
**Trait**	**units**	**n**	**min**	**mean**	**sd**	**max**	**n**	**min**	**mean**	**sd**	**max**
Feeding relocation strategy											
endocoprid	%	29	0.00	0.00	0.00	0.00	32	0.00	0.84	0.27	1.00
paracoprid	%	29	0.00	0.76	0.44	1.00	32	0.00	0.16	0.27	1.00
telecoprid	%	29	0.00	0.24	0.44	1.00	32	0.00	0.00	0.00	0.00
Adult trophic preference											
saprophagous	%	29	0.00	0.02	0.09	0.50	32	0.00	0.30	0.39	1.00
coprophagous	%	29	0.50	0.92	0.12	1.00	32	0.00	0.68	0.39	1.00
micophagous	%	29	0.00	0.00	0.00	0.00	32	0.00	0.00	0.02	0.10
necrophagous	%	29	0.00	0.06	0.09	0.20	32	0.00	0.02	0.05	0.20
Dry biomass	mg	29	3.620	111.036	144.954	579.860	29	0.500	2.549	2.188	8.880
Fresh weight	mg	29	13.980	478.854	657.297	2597.300	29	1.000	8.505	7.893	30.000
Body length	mm	29	5.213	14.127	7.711	31.306	29	3.205	5.222	1.560	8.447
Head length	mm	29	1.203	3.154	1.638	6.904	29	0.557	0.943	0.281	1.525
Head width	mm	29	1.638	4.191	2.501	10.466	29	0.838	1.356	0.466	2.258
Pronotum height	mm	29	1.513	3.876	1.920	7.999	29	0.698	1.236	0.334	1.869
Pronotum length	mm	29	1.969	4.892	2.317	9.425	29	0.844	1.412	0.452	2.445
Pronotum width	mm	29	2.762	7.437	4.034	16.685	29	1.130	1.923	0.635	3.392
Abdomen height	mm	29	2.121	5.309	2.741	11.389	29	0.958	1.689	0.562	2.607
Elytrum area	mm²	29	3.092	27.983	31.964	126.458	29	1.062	3.164	1.820	6.495
Elytra length	mm	29	2.040	6.081	3.755	14.977	29	1.804	2.867	0.827	4.476
Elytra width	mm	29	1.565	3.744	2.041	8.980	29	0.605	1.152	0.364	1.910
Elytrum weight	mg	29	0.280	5.287	7.108	28.540	29	0.000	0.216	0.196	0.660
Protibia area	mm²	29	0.357	4.257	5.256	20.708	29	0.067	0.212	0.152	0.568
Protibia length	mm	29	1.080	3.666	2.573	10.144	29	0.367	0.663	0.239	1.110
Protibia tooth length	mm	29	0.356	0.995	0.561	2.391	29	0.216	0.378	0.145	0.723
Metatibia area	mm²	29	0.240	2.669	2.970	11.398	28	0.041	0.166	0.125	0.529
Metatibia length straight	mm	29	1.140	3.752	2.971	11.764	28	0.470	0.952	0.353	1.726
Metatibia length curved	mm	29	1.150	3.811	3.040	11.983	28	0.472	0.955	0.355	1.725
Wing area	mm²	29	8.597	70.052	74.817	264.918	27	3.363	13.046	8.676	42.505
Wing length	mm	29	5.287	13.787	7.448	27.757	27	3.230	5.880	1.823	10.064
Wing load	mg/mm²	29	0.346	1.199	0.559	2.189	27	0.092	0.200	0.105	0.566

Table S3. Results of Mantel (Spearman-rank) correlation tests between phylogenetic pairwise site dissimilarity and aridity for the wet and dry seasons in the two sampled years. Significance levels after Bonferroni correction: * p < 0.0125; *m.s.* = marginally significant.

Survey campaign	N	rho	*p*
Wet 2013	45	0.4440	0.011 *
Wet 2014	45	0.5794	0.005 *
Dry 2013	45	0.3610	0.031 *m.s.*
Dry 2014	45	0.4697	0.004 *

Table S4. Mantel correlations and *p-values* between the three aspects of beta diversity (taxonomic, functional and phylogenetic) of dung beetle communities in the wet and dry seasons along an aridity gradient from the Mediterranean Sea to the Sahara Desert during two consecutive years. Total beta diversity and its nestedness and turnover partitions are shown. Whole triangular distance matrices were used separately for the three partitions of beta diversity (i.e. total, turnover and nestedness partitions). ns: non significant; **p*<0.01; **p*<0.001.

Beta	Campaign	Taxonomic vs. Phylogenetic		Taxonomic vs. Functional		Phylogenetic vs. Functional	
**total**	dry2013	0.945	**	0.466	*	0.548	**
	dry2014	0.893	**	0.489	*	0.419	*
	wet2013	0.972	**	0.732	**	0.777	**
	wet2014	0.925	**	0.634	**	0.588	*
**nestedness**	dry2013	0.956	**	0.678	**	0.757	**
	dry2014	0.672	**	0.165	ns	-0.113	ns
	wet2013	0.798	**	-0.103	ns	-0.265	ns
	wet2014	0.690	**	-0.003	ns	-0.219	ns
**turnover**	dry2013	0.973	**	0.739	**	0.768	**
	dry2014	0.774	**	0.524	**	0.453	*
	wet2013	0.929	**	0.717	*	0.612	*
	wet2014	0.940	**	0.569	**	0.483	*

Table S5. Results of multiple analysis of variance (MANOVA) between sparse IPCA components for the wet and the dry seasons and aridity. Significance levels after Bonferroni correction: * p < 0.0125

	**Coefficient ± S.E.**	**d.f.**	**F**	***p***
Wet season				
Component 1	7.493 ± 1.884	1, 8	15.820	0.0041 *
Component 2	-0.033 ± 3.251	1, 8	0.0001	0.992
Dry season				
Component 1	7.209 ± 2.018	1, 8	12.760	0.0073 *
Component 2	-2.475 ± 3.131	1, 8	0.625	0.452
